# Indocyanine green combined with autologous blood and methylene blue for pulmonary nodules localization in 272 cases: a novel localization method

**DOI:** 10.1007/s13304-025-02350-7

**Published:** 2025-08-11

**Authors:** Quan Du, Zijie Wang, Jian Chen, Hong Chen, Yuanrong Tu, Min Lin, Jianfeng Chen

**Affiliations:** 1 The Graduate School of Fujian Medical University, Fuzhou, 350122 China; 2 Department of Thoracic Surgery, The First Affiliated Hospital, Fujian Medical University, Fuzhou, 350005 China; 3 Department of Thoracic Surgery, National Regional Medical Center, Binhai Campus of The First Affiliated Hospital, Fujian Medical University, Fuzhou, 350212 China; 4 Department of Interventional Radiology, The First Affiliated Hospital, Fujian Medical University, Fuzhou, 350005 China; 5 Department of Interventional Radiology, National Regional Medical Center, Binhai Campus of the First Affiliated Hospital, Fujian Medical University, Fuzhou, 350212 China

**Keywords:** Pulmonary nodule, Localization, Wedge resection, Indocyanine green, Video-assisted thoracoscopic surgery

## Abstract

To explore the feasibility and safety of using indocyanine green combined with autologous blood and methylene blue for the localization of small pulmonary nodules in thoracoscopic wedge resection. Patients who met the inclusion criteria underwent CT-guided percutaneous lung puncture injection of localization agents to locate pulmonary nodules at our center from November 2023 to September 2024. Under thoracoscopy, pulmonary nodules were visualized, and wedge resection was performed. The feasibility was preliminarily verified by evaluating whether the localization agent concentrated around the nodules and help surgeons successfully completed wedge resection under marker guidance. The safety was verified by the incidence of adverse reactions during puncture and surgery. A total of 272 patients were included, comprising 110 males and 162 females, with an average age of 50.60 (14.17) years. In all cases except the first four, the localization agent concentrated around the nodules, achieving precise localization of pulmonary nodules. Nodules were found in all resected tissues, with negative margins. In 4 patients, excessive localization agent dosage caused marker diffusion and pleural staining. The overall localization success rate was 98.53%, and when the injection volume of the localization agent was 0.2–0.5 mL, the localization success rate was 100.0%. All cases successfully completed thoracoscopic wedge resection, and nodule lesions were found with negative margins. Indocyanine green combined with autologous blood and methylene blue for the localization of small pulmonary nodules is safe and feasible in lung wedge resection.

## Introduction

Lung cancer is the second most common malignancy in humans and the leading cause of cancer-related deaths worldwide [1]. Thanks to the increased awareness of physical examinations for pulmonary diseases after the COVID-19 pandemic, along with the implementation of low-dose computed tomography (CT), the diagnosis of pulmonary nodules, which are considered malignancies in the early stage, has increased significantly. For patients with small-sized early-stage lung cancer, surgery is the best option to ensure long-term survival. It can increase the 5-year survival rate ten-fold compared to patients who do not undergo surgery [2]. The standard surgical procedure is lobectomy or segmentectomy. A recent randomized-controlled study shows that there were no significant differences between lobectomy and segmentectomy in mortality rate and complications in patients who have invasive peripheral non-small cell lung cancer (NSCLC) with a diameter ≤ 2 cm [3]. Importantly, there were no significant differences in overall survival (OS) rates between segmentectomy and wedge resection in patients with stage Ia NSCLC with tumor size ≤ 2 cm [4]. According to this, sub-lobar resection in patients with a peripheral nodule size ≤ 2 cm is recommended.

However, small pulmonary nodules are often invisible under thoracoscopy and difficult to palpate [5], increasing the risk of failure in wedge resection. To achieve minimally invasive surgery, preoperative localization may decrease the conversion to thoracotomy or even surgical failure [6]. Dye marking was considered a safe and efficient way to localize pulmonary nodules. Indocyanine green (ICG) is a near-infrared imaging reagent currently approved by the US Food and Drug Administration (FDA) for clinical use. It demonstrates better stability, fewer complications, and visibility of pulmonary nodules under a fluorescence endoscopy system according to the enhanced permeability and retention (EPR) effect [7]. As a water-soluble molecule, it is easy to diffuse throughout a wide range of lung parenchyma.

An ICG-human albumin mixture that reduces the risk of marker displacement or migration has been reported [8]. Autologous blood is rich in albumin at no extra cost. By mixing ICG with autologous blood it will be an economical and safe way to alleviate ICG diffusion. Methylene blue was introduced into the mixture in case of the failure of ICG localization. The dual protection scheme will increase the success rate of localization and the reliability of the operation. Since then, we have tried to use ICG mixed with autologous blood and methylene blue for preoperative CT-guided percutaneous lung localization of small pulmonary nodules and masses in our department, and good results have been achieved.

## Methods

### Subject

A total of 272 patients with pulmonary nodules and masses diagnosed by chest CT in the Department of Thoracic Surgery, the First Affiliated Hospital of Fujian Medical University, from November 2023 to September 2024 were collected, including 110 males and 162 females. Inclusion criteria were as follows: 1. Chest CT scan showed round or irregular density increased in the lung with a diameter ≥ 5 mm and ≤ 30 mm, and the nodules grew during follow-up. 2. The distance from the lesion to the pleura ≤ 2 cm or the nodule is located in the outer 1/3 of the lung. 3. The pulmonary nodule size ≤ 2 cm. 4. The patients met the indications of wedge resection and had no surgical contraindications. Exclusion criteria included patients with pulmonary vascular lesions, lesions close to major pulmonary vessels, severe cardiopulmonary insufficiency, or bleeding tendency. All procedures performed in studies involving human participants were in accordance with the ethical standards of the institutional and/or national research committee and with the 1964 Helsinki Declaration and its later amendments or comparable ethical standards. The study was approved by the Bioethics Committee of the First Affiliated Hospital of Fujian Medical University (No. [2024] 578). Informed consent was obtained from all individual participants included in the study. Additional informed consent was obtained from all individual participants for whom identifying information is included in this article.

### Localization procedure

#### Reagents and equipment

4 K fluorescence endoscopy system UX5 (Mindray Medical, China), indocyanine green, and methylene blue. 25 mg ICG powder was dissolved in 10 ml sterile water to form a 2.5 mg/mL ICG solution. Milliliters of ICG solution were mixed with autologous blood and methylene blue to form 1 mg/mL, 0.5 mg/mL, 0.25 mg/mL, and 0.05 mg/mL localization agents. The volume of ICG depended on the concentration of ICG we tested.

#### Introduction of localization agent

**Fig. 1:**
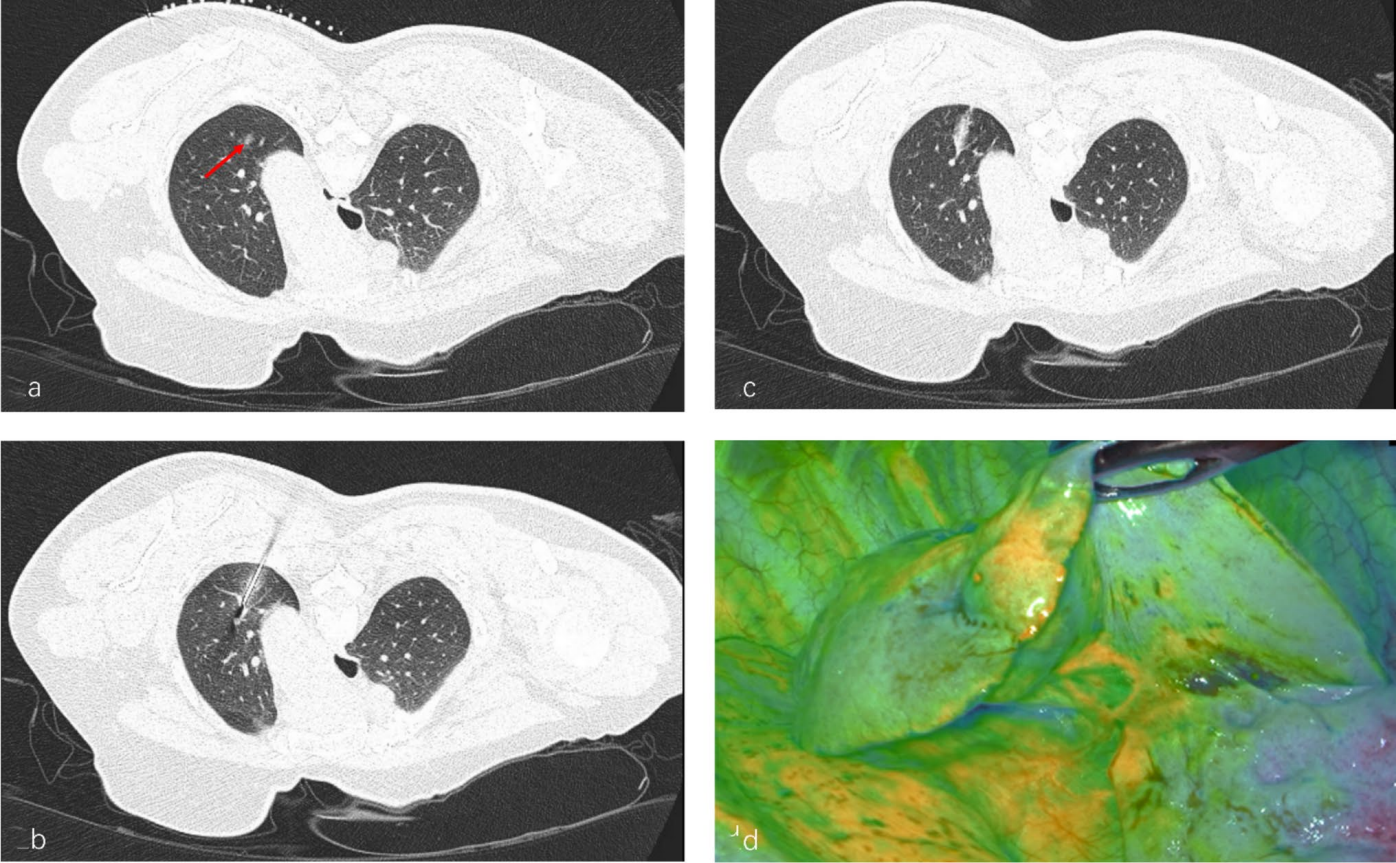
CT-guided puncture localization agent injection and over dose of localization agent stained the pleura and failed to localize the nodule (1a: the arrow indicates the pulmonary nodule; 1b: the needle reached the injection site; 1c: localization agent injection complete; 1d: Excessive use of the localization agent resulted in pleural staining and localization failure.)

The appropriate position was determined according to the location of the nodule in the CT room. The 18-G PTC needle (Hakko Co., Ltd, Japan) was inserted at a 30°–45° angle to the skin surface, gradually advanced into or within 1 cm of the lesion under continuous CT fluoroscopic guidance. After confirming the needle tip position, the needle was slightly withdrawn to verify the absence of bronchial or vascular entry via CT scan (Siemens SOMATOM Definition AS, Germany). Approximately 0.1–1.0 mL of the localization agent was then injected at a constant speed of 0.1 mL per second. The needle was carefully removed at the end of the injection procedure. Postoperative CT scanning was performed to assess the adequacy of marker distribution and exclude complications such as pneumothorax or hemothorax. (Fig. 1a–c).

#### Localization agent dose optimization protocol

The mixed localization agent with indocyanine green at a concentration of 0.05 mg/mL was divided into concentration gradients of 1 mL, 0.9 mL, 0.8 mL, 0.7 mL, 0.6 mL, 0.5 mL, 0.4 mL, 0.3 mL, and 0.2 mL. The localization-surgery interval was controlled at approximately 2 h. During the operation, an evaluation team composed of two experienced surgeons independently scored the localization efficacy of each case according to a unified rating scale, a score higher than 2 points is considered a successful localization. The rating scale is as follows:

1 point: Severe diffusion or pleural staining, relies on CT images and clinical experience, provides no assistance to surgeons.

2 points: Relatively clear with relatively sharp boundaries, mild diffusion, requires reference to CT images.

3 points: Clearly visible with sharp boundaries, no diffusion, greatly assists surgeons.

### Surgical procedure

Patients were brought to the operating room less than 24 h after localization. All patients underwent single-utility port thoracoscopic surgery with double-lumen endotracheal intubation under general anesthesia and one-lung ventilation. The thoracoscopic hole was made at the midaxillary line of the seventh intercostal space, and the main operation hole was made at the anterior axillary line of the fourth intercostal space. Thoracoscopic exploration was performed, and a green plaque demonstrated the localization area under fluorescence mode. A wedge resection was performed, and a linear cutting closed suture device was used to perform a wedge resection according to the fluorescence mark. The specimen was carefully searched according to the puncture path, and the frozen section was sent during the operation to confirm the posterior line.

### Observation indicators

Operation time, localization-operation time interval, the concentration of indocyanine green, the dose of localization agent, resection margin status, the distance from the cutting edge of the anastomotic nail to the nodule, postoperative hospital stay, extubation time, drainage volume, and complications were collected. Localization success rate: If the localization is successful, the fluorescent signal in the lung nodule will be intensely concentrated at the site of the nodule, with a clear contrast to normal lung tissue. If diffusion occurs, the fluorescent signal will be distributed over a large area of lung tissue, even contaminating the pleural membrane.

### Statistical analysis

Patients meeting the inclusion and exclusion criteria in outpatient settings were enrolled via convenience sampling. Using SPSS 25.0 to conduct the Shapiro–Wilk normality test. Normal distribution of quantitative data is described by the mean (standard deviation), while skewed distribution is described by the M (P25, P75). Count data is described by frequency and percentage.

## Results

### Characteristics of patients

This study collected 272 patients who met the inclusion criteria and underwent this new method for the localization of pulmonary nodules from November 2023 to September 2024 at our center. There were 110 males and 162 females with an average age of 50.60 (14.17) years, and the average body mass index (BMI) was 23.28 (3.10). There was a history of smoking in 38 cases. All patients had no family history of lung cancer. There were 83 patients with risk factors, including 68 with hypertension, 19 with diabetes, 8 with coronary heart disease, and 4 with hepatitis B cirrhosis (Table 1). A patient may have multiple risk factors. No patient data were missing in this study.

**Table 1:** General characteristic of patients

Characteristic	n = 272
Male: Female	110: 162
Age (years), mean (SD)	50.60(14.17)
BMI, mean (SD)	23.28(3.10)
Smoking history	
Yes (n%)	38 (13.97%)
No (n%)	234 (86.03%)
Family history	
Yes (n%)	0 (0%)
No (n%)	272 (100%)
Risk factors	83 (30.51%)
Hypertension	68
Diabetes	19
Cardiovascular disease	8
Hepatitis B cirrhosis	4
No risk factors	189 (69.49%)

### Characteristics of pulmonary nodules

In the 272 cases, a total of 310 pulmonary small nodules were treated, including 167 pure ground glass nodules, 91 mixed ground glass nodules, and 52 solid nodules. The median diameter of nodules was 6.00 (5.00, 9.00) mm for the longest diameter, and 5.00 (4.00, 5.00) mm for the shortest diameter. The median distance from the nodule to the visceral pleura was 6.25 (0.00, 11.91) mm. The distribution of nodules and intraoperative frozen section results are shown in Table 2.

**Table 2:** Characteristic of pulmonary nodules

Characteristic	n = 310
Long diameter of nodule (mm), M (P25, P75)	6.00(5.00, 9.00)
Short diameter of nodule (mm), M (P25, P75)	5.00(4.00, 5.00)
Depth of nodule (mm), M (P25, P75)	6.25(0.00, 11.91)
Radiological type	
pGGO (n%)	167 (53.87%)
mGGO (n%)	91 (29.35%)
Solid (n%)	52 (16.77%)
Location	
Upper right (n%)	128 (41.30%)
Middle right (n%)	19 (6.13%)
Lower right (n%)	49 (15.81%)
Upper left (n%)	68 (21.93%)
Lower left (n%)	46 (14.83%)
Pathological type	
Minimally invasive adenocarcinoma (n%)	214 (69.03%)
Adenocarcinoma in situ (n%)	28 (9.03%)
Benign nodule (n%)	68 (21.94%)

### Operation data

The median operation duration was 70 (58.50, 78.75) minutes. The median amount of intraoperative bleeding was 50.00 (30.00, 50.00) mL. The distances from the suture clip margins to the nodule all met the standard, with a median distance of 1.2 (0.80, 2.00) cm, and all margins were negative. No patients experienced puncture or surgery-related complications, and the median length of hospital stay was 4.00 (3.00, 5.00) days, the median duration of tube removal was 3.00 (2.00, 4.00) days, and the median amount of closed chest drainage was 375 (232.5, 542.5) mL (Table 3).

**Table 3:** Intraoperative and postoperative data

Data	n = 310
Surgery duration (min), M (P25, P75)	70(58.50, 78.75)
Intraoperative bleeding (mL), M (P25, P75)	50.00 (30.00, 50.00)
The distance from the cutting edge of the anastomotic nail to the nodule (cm), M (P25, P75)	1.2 (0.80, 2.00)
Resection margin	
Negative (n%)	310 (100%)
Positive (n%)	0 (0%)
Postoperative hospital stay (d), M (P25, P75)	4.00 (3.00,5.00)
Extubation time (d), M (P25, P75)	3.00(2.00, 4.00)
Drainage volume (mL) M (P25, P75)	375 (232.5,542.5)

### Localization

**Fig. 2:**
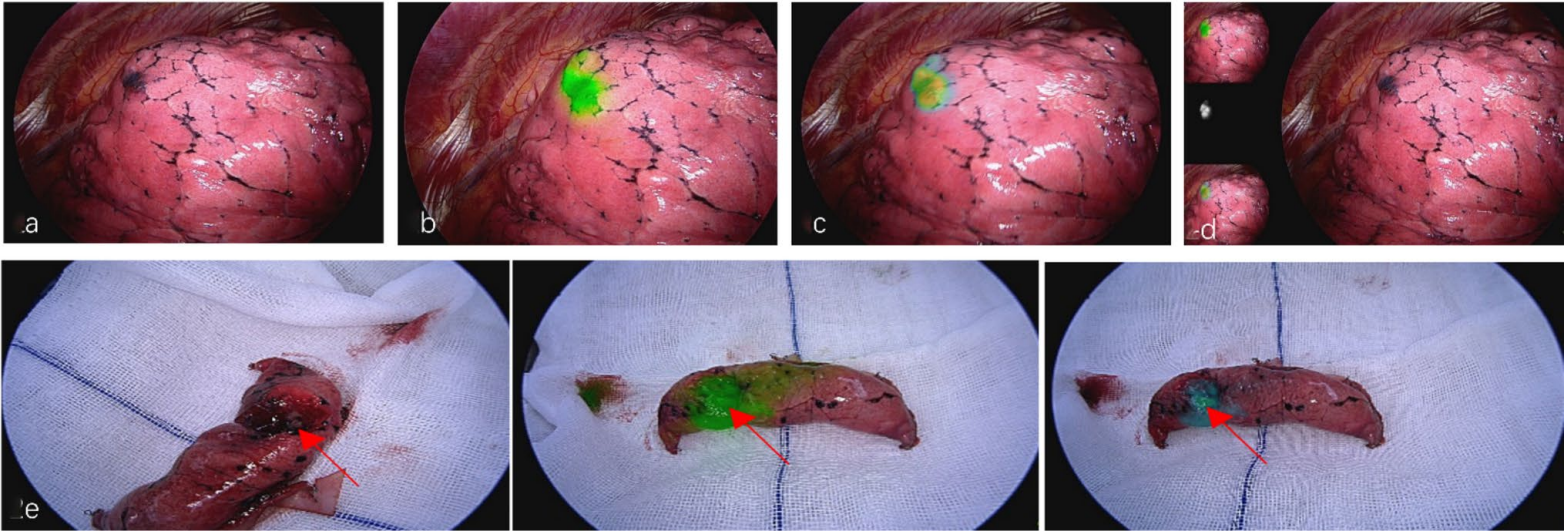
Successful localization under 4 K fluorescence endoscopic system during surgery and after wedge resection (**a**: dark purple ecchymosis sign made by methylene blue was found under white light mode; **b**: a concentrated fluorescence signal was found under fluorescence mode; **c**: warm color area indicating more likely where the nodule is under color fluorescence mode; **d**: multiple mode imaging; 2e: the arrow indicates the nodule after wedge resection.)

All cases were subjected to CT-guided lung puncture injection of the localization agent before surgery, without any complications such as pneumothorax or hemothorax. In cases 1, 2, 3, and 4, the localization agent dose was reduced from 1 to 0.6 mL, and all showed diffuse uptake in the chest cavity under fluorescence mode. Thus, the scores given by both surgeons were both ≤ 2 points. In case 1, the lung tissue was squeezed during surgery, causing the localization agent to leak from the puncture site, staining the pleura (Fig. 1d). In the rest of the cases, the localization agent dose was reduced from 0.5 to 0.2 mL, and all showed a focal uptake near the nodule, resulting in successful localization (Fig. 2). When the dosage of the localization agent was determined, adjusting the localization-surgery interval from 1 to 24 h and varying ICG concentrations resulted in all localization marker efficacy scores being higher than 2 points, providing definite assistance to surgeons. The localization agent dose and the concentration of indocyanine green were selected based on the experience of the first 20 cases, and all showed a focal uptake near the nodule, with a small area of diffusion after squeezing lung tissue, resulting in successful localization. Due to the different locations of the nodules in each case and the uncontrollable differences in localization techniques, there were differences in localization effects under the same conditions of indocyanine green concentration, localization agent dose, and interval between localization and surgery, but these differences did not affect the precise localization and resection of the nodules. The key determinant of successful localization is the dosage of the localization agent. Lower ICG concentrations can be used when considering cost-effectiveness, and the wide range of localization-surgery intervals allows for high flexibility in clinical application. The final formula of the localization agent is 4 mL of autologous blood, 0.9 mL methylene blue, and 0.1 mL ICG solution with the ICG concentration of 0.05 mg/mL. The best dosage of the localization agent is 0.2 mL for surgery day localization or 0.5 mL for the day before surgery (Table 4).

**Table 4:** Dosage optimization score

Dosage	Score 1	Score 2	Average
1 mL	1	1	1
0.9 mL	1	1	1
0.8 mL	1	1	1
0.7 mL	1	2	1.5
0.6 mL	2	1	1.5
0.5 mL	2	3	2.5
0.4 mL	3	3	3
0.3 mL	3	3	3
0.2 mL	3	3	3

## Discussion

**Fig. 3:**
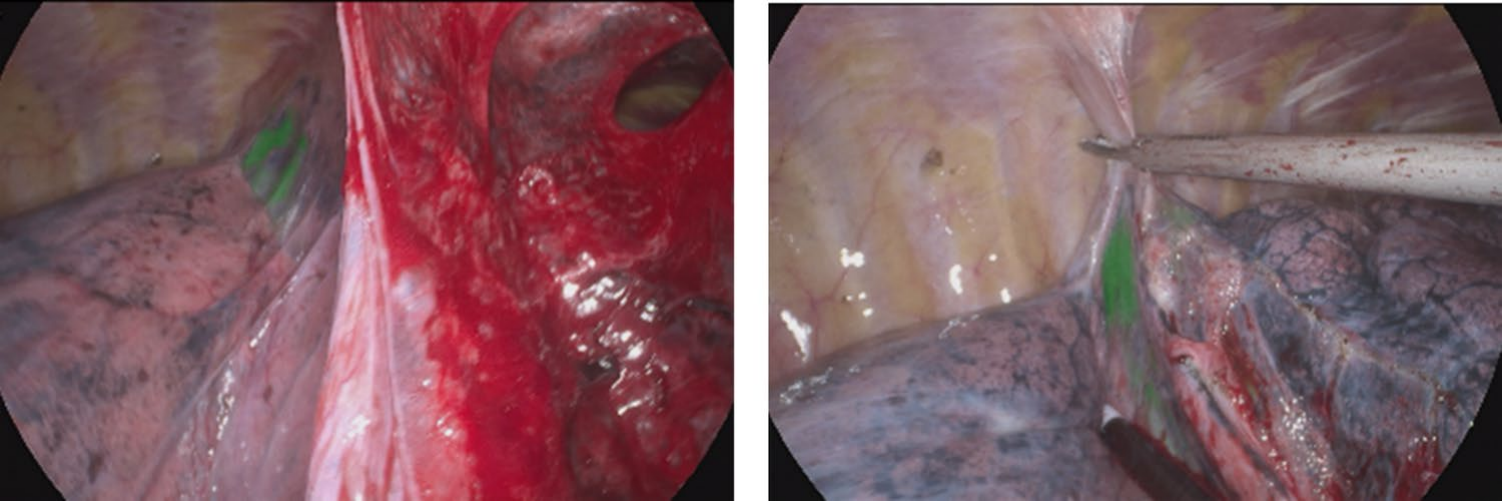
The dispersion of the localization agent was not serious when the pleural adhesions were released

Recent clinical trials have redefined surgical strategies for early-stage peripheral non-small cell lung cancer (NSCLC), with sub-lobar resection increasingly favored for appropriately selected cases. The landmark JCOG0804/WJOG4507L trial demonstrated a 99.7% relapse-free survival rate for peripheral adenocarcinomas ≤ 2 cm in size with a consolidation-to-tumor ratio (CTR) ≤ 0.25 and no nodal involvement. Complementing these findings, the phase III JCOG0802/WJOG4607L trial further established sub-lobar resection as superior to lobectomy in 5-year overall survival while preserving pulmonary function (FEV1/FVC), despite comparable recurrence rates. These advancements underscore the critical role of precise nodule localization, particularly for non-palpable lesions, to ensure adequate resection margins while minimizing parenchymal loss (Fig. 3).

Current localization techniques exhibit distinct limitations. CT-guided hook wire placement, though reliable, carries risks of wire displacement (20–24%) and complications such as pneumothorax (10–35%) or hemothorax (5–15%) [9]. Notably, ICG localization has demonstrated advantages in procedural efficiency, reduced patient discomfort, and scheduling flexibility compared to hook wire methods. Percutaneous injection of liquid markers (e.g., lipiodol, methylene blue, or ICG) remains widely adopted for its simplicity but requires operator expertise to mitigate complication risks [10]. In our study, this approach achieved successful localization without complications. Contrastingly, electromagnetic navigation bronchoscopy (ENB) reduces radiation exposure and procedural pain under general anesthesia but shows limited accuracy (89% vs. 98.3% for CT-guided methods) and poor peripheral lesion accessibility, compounded by higher costs [11]. Emerging alternatives like ICG inhalation offer non-invasive segmental-level localization but face challenges in agent retention due to anatomical variability [12, 13]. Studies have attempted to use a mixture of ICG and lipiodol to prepare an emulsion, whose purpose, similar to that of this study, is to extend the localization marking duration and alleviate the diffusion of localization markers. Lipiodol can also appear as a high-density shadow under X-ray, allowing real-time imaging localization with a C-arm machine during surgery, which adds an extra guarantee for localizing pulmonary small nodules. However, the consistency of the mixture is difficult to ensure. The mixture of indocyanine green and lipiodol by different operators may vary, failing to guarantee the quality of the localization agent and potentially leading to localization failure. The author mentioned that the localization marker can be maintained for up to 6 days, and also reported one case of pneumonia. The risk of vascular embolism should also be considered. Further verification is needed for safety and technical generalization [14].

Methylene blue, an early liquid marker, enables rapid visualization but tends to diffuse rapidly (requiring surgery within 4 h) and causes ambiguous staining in patients with pre-existing lung pigmentation (e.g., smokers or those with silicosis), leading to suboptimal success rates (≈85%) [15]. Methylene blue localization has no significant impact on the pathological diagnostic accuracy of intraoperative frozen sections (with an accuracy rate of over 90%), but high-dose injection (> 2 ml) may reduce section quality by approximately 15%. Such issues do not exist with ICG [16]. Autologous blood localization addresses safety concerns with minimal foreign body reactions and extended duration (up to 12 h via viscous hematoma formation) [17], though low tissue contrast limits its utility in pigmented lungs [18]. ICG emerges as a versatile alternative detectable via fluorescence imaging, unaffected by surface pigmentation. Its enhanced permeability and retention (EPR) effect facilitates tumor-specific accumulation, resulting in distinct intraoperative color contrasts (warm-toned lesions vs. cool parenchyma under fluorescence). However, rapid diffusion, akin to other liquid markers remains a limitation.

To overcome these constraints, we developed a composite agent combining ICG, autologous blood, and methylene blue. This hybrid approach synergizes ICG’s fluorescence precision, autologous blood’s diffusion-delaying viscosity, and methylene blue’s fail-safe visual marking. Initial trials revealed that doses > 0.5 mL caused pleural staining during tissue manipulation, though methylene blue ensured surgical guidance. Optimized doses (0.2–0.5 mL) demonstrated stable localization with minimal diffusion, particularly in ground-glass nodules (GGNs), where the agent concentrated visibly under fluorescence. In solid nodules, localization primarily occurred at the puncture site, suggesting vascular dynamics influence agent distribution. The composite marker maintained efficacy for ≤ 24 h and resisted pleural contamination during adhesion dissection—a marked improvement over methylene blue alone [19].

While this pilot study confirms feasibility and safety, limitations include heterogeneous nodule subtypes (imaging/pathological) and lack of comparative controls. Future randomized trials should evaluate clinical advantages against established methods. Nevertheless, the integration of fluorescence targeting, prolonged stability, and dual marking mechanisms positions this approach as a promising tool for precision-guided resection of small peripheral lung nodules.

## Conclusions

The ICG-autologous blood-methylene blue composite represents an innovative localization strategy for peripheral pulmonary nodules (≤ 2 cm from pleura, ≤ 2 cm diameter) undergoing CT-guided wedge resection. By harmonizing fluorescence precision, delayed diffusion, and visual confirmation, this method addresses key limitations of existing techniques. Further validation through controlled trials will clarify its broader applicability.

## Data Availability

The datasets generated during and/or analysed during the current study are not publicly available due privacy concerns but are available from the corresponding author on reasonable request.
